# Integrated bulk and single-cell transcriptomics reveals TREH as a novel protective biomarker and prognostic predictor in clear cell renal cell carcinoma

**DOI:** 10.3389/fonc.2026.1808369

**Published:** 2026-05-11

**Authors:** Kuo Ma, Yanan Wang, Xiangdong Xue, Junling Hu, Lei Li

**Affiliations:** 1 Department of Urology, The First Affiliated Hospital of Henan Medical University, Xinxiang, China; 2 Department of Medical Oncology, The First Affiliated Hospital of Henan Medical University, Xinxiang, China

**Keywords:** biomarkers, clear cell renal cell carcinoma, epithelial-mesenchymal transition, multiple omics, TREH, tumor microenvironment

## Abstract

**Background:**

Clear cell renal cell carcinoma (ccRCC) is marked by substantial intratumoral heterogeneity and highly variable clinical outcomes. The identification of robust biomarkers that drive tumor progression is essential for improving prognostic accuracy and guiding therapeutic strategies.

**Methods:**

We employed an integrated transcriptomic data analysis strategy, combining Weighted Gene Co-expression Network Analysis (WGCNA) with machine learning algorithms (LASSO and GBM). To screen for prognostic candidates, the TCGA-KIRC cohort was allocated into a 70% training set for model development and a 30% testing set for internal validation. The clinical relevance, immunological landscape, and biological functions of the top candidate were systematically characterized using scRNA-seq analysis and validated through extensive *in vitro* and *in vivo* assays.

**Results:**

Trehalase (TREH) was identified as a novel, independent protective factor for ccRCC. Low TREH expression correlated significantly with advanced pathologic stages, Fuhrman histological grade, distant metastasis, and poor survival outcomes (OS, DSS, and PFI). A prognostic nomogram integrating TREH expression with clinical parameters demonstrated superior predictive accuracy. At the single-cell level, TREH was predominantly and specifically expressed in epithelial cells. Notably, computational analysis predicted that TREH-deficient epithelial cells may engage in more intensive pro-tumorigenic and angiogenic crosstalk with the microenvironment, particularly through the VEGF, PLG, and APP signaling pathways. Mechanistically, our data suggest that TREH acts as a tumor suppressor. Downregulation of TREH is associated with EMT promotion and an immunosuppressive microenvironment. The causal link between TREH enzymatic activity and these phenotypes remains to be further defined. In addition, TREH overexpression significantly inhibited cell proliferation, migration, and invasion *in vitro* and suppressed tumor growth *in vivo*.

**Conclusion:**

Our study establishes TREH as a robust prognostic biomarker and a functional tumor suppressor in ccRCC. The loss of TREH is associated with tumor progression and correlates with EMT activation and a pro-angiogenic microenvironment, suggesting its potential as a therapeutic target for improving patient outcomes.

## Methods

### Bulk transcriptome analysis

Gene expression profiles and corresponding clinical data for Kidney Renal Clear Cell Carcinoma (KIRC) were obtained from The Cancer Genome Atlas (TCGA) database. To identify key prognostic molecules, we employed an integrated analytical workflow. First, Weighted Gene Co-expression Network Analysis (WGCNA) was performed, and genes from the top three modules most negatively correlated with tumor status were selected. These genes were then intersected with downregulated differentially expressed genes (DEGs) identified in the TCGA-KIRC cohort. Univariate Cox regression analysis of this intersecting set yielded 13 candidate genes associated with favorable prognosis. To refine the selection, two machine learning algorithms—Gradient Boosting Machine (GBM) and Least Absolute Shrinkage and Selection Operator (LASSO) regression—were applied (1, 2). The top 10 prognostic genes from each method were retained, and their overlap was determined. Multivariate Cox proportional hazards regression was subsequently performed on this overlapping gene set to establish a robust prognostic signature, from which TREH was selected for further investigation. The prognostic value of TREH expression was evaluated using Kaplan-Meier survival analysis and multivariable Cox proportional hazards regression models. The association between TREH expression levels and key clinicopathological parameters was also statistically assessed.

### Single-cell transcriptome analysis

We acquired the scRNA-seq dataset GSE242299 from the GEO database, which consists of eight tumor samples and eight paired adjacent non-malignant tissues from ccRCC patients. All data quality control and downstream analyses were carried out via the Seurat R package (v5.3.0). Low-quality cells were filtered out using the following criteria: cells with fewer than 300 or more than 7,000 detected genes, cells where mitochondrial genes accounted for more than 10% of total counts, cells with hemoglobin gene expression exceeding 3%, and cells with total RNA counts less than 1,000 or greater than 20,000. After quality control, a total of 18,402 cells and 33,538 genes were retained for downstream analysis. Standard procedures including data normalization, scaling, principal component analysis (PCA), and graph-based clustering were applied. Cell types were annotated using well-established canonical markers. Intercellular communication analysis was conducted using the R package CellChat to infer and visualize differential signaling pathways between epithelial cells derived from TREH-high and TREH-low expressing tumor tissues.

### Cell culture and transfection

Human RCC cell lines were obtained from Chinese Academy of Sciences (Shanghai). HK-2, ACHN, A-498 Cells were all cultured in MEM medium (Gibco, China),OS-RC-2, 769-P, 786-O Cells were all cultured in RPMI 1640 medium (Gibco, China), CAKI-1Cell was cultured in McCoy’s 5a+10% FBS + 1% P/S supplemented with 10% fetal bovine serum (FBS, Gibco, USA), and maintained in a humidified incubator at 37 °C with 5% CO2. Cell lines stably overexpressing TREH and knocking down TREH were constructed, and human TREH regulatory lentiviral vectors (Lv-TREH, Lv-shTREH) and corresponding control vectors (Lv-NC) were obtained from Jikai Biotechnology. OS-RC-2, CAKI-1, 786-O, and 769P cells were transfected by Lipofectamine 3000 (Invitrogen, USA). Cells were collected 48 h after transfection for experimental analysis.

### Wound-healing assay

Ransfected cells were inoculated into six-well plates at a density of 30×10^4^ and incubated until a confluent monolayer formed. A linear wound was created on the well surface using a 10μL pipette tip, after which the wells were rinsed with PBS. Subsequently, the cultures were maintained in serum-free medium for a duration of 24 hours. Images of the gap were captured using a microscope and camera system at the 0-hour baseline and the 24-hour endpoint. The healing area of the scratch was calculated by ImageJ using the formula: healing rate =(0h area -24h area)/0h area ×100%.

### Migration and invasion assay

For Transwell migration assay, the transfected cells were seeded in serum-free medium (5×10^4^cells/well) in the upper chamber of the Transwell system and 10% FBS in the lower chamber. During the invasion assays, Matrigel-coated (BD Biosciences, San Joe, CA,USA) transwell chambers were used to examine the invasion ability of cells. The following procedures were similar to those used for the migration assays. After a 24-48h incubation time, the cells on the bottom surface of the filter membranes were fixed, stained and counted under a light microscope(Five fields were randomly selected from each visual field and counted and averaged).

### Colony formation assay

Transfected cells (1×10^3^ per well) were plated in 6-well plates for clonogenic analysis. The cell culture medium was changed every 3 days. After two weeks, the medium was removed and the cells were fixed with 4% paraformaldehyde. Finally, the colonies were stained using a 0.5% crystal violet solution(Colony formation rate = number of positive clones/number of inoculated cells×100%).

### *In vivo* tumor xenograft model

To establish xenografts, 4- to 6-week-old male BALB/c nude mice were obtained from Vital River Laboratories (Beijing) and maintained under SPF standards with ethical approval. We randomly divided the mice and injected them subcutaneously with 6×10^6^ cells. Prior to injection, OS-RC-2 and 786-O cells were transfected with LV-TREH/vector or sh-TREH/control constructs. The cells were prepared in 50 µL PBS combined with 50 µL Matrigel (Corning). We monitored tumor growth by taking measurements every three days. Formulas for calculating tumor volume (V = length × width²/2), methods for measuring tumor weight (stripping the tumor after neck removal and weighing it with electronic balance), criteria for determining the end point of the experiment (stopping the experiment when the tumor volume reaches 2000mm³).

### Western blot

Cell lysates were prepared using RIPA buffer mixed with inhibitor cocktails for proteases and phosphatases. Protein samples were resolved by SDS-PAGE and electrotransferred onto nitrocellulose membranes. Subsequently, the blots were probed with specific primary antibodies overnight at 4°C, we applied IRDye 800CW secondary antibodies (LI-COR, USA). The specific bands were visualized utilizing the Odyssey^®^ Imaging System.

### Statistical analysis

All statistical analyses performed in this study relied on R software (v4.1.3) and GraphPad Prism 10. The significance of intergroup differences was determined via T-test and Wilcoxon rank-sum test, and Spearman’s correlation coefficient was applied for correlation analyses. All *in vitro* functional experiments were marked as independently repeated 3 times, with 3 replicate wells set up each time. Statistical significance was defined as *P < 0.05, **P < 0.01,***P < 0.001.

## Introduction

Kidney cancer is among the ten most common cancers worldwide (3, 4), with clear cell renal cell carcinoma (ccRCC) representing the predominant histologic subtype, accounting for approximately 75% of all cases (5). Despite advances in surgical management for localized disease, the clinical outcomes of ccRCC remain highly variable due to its profound heterogeneity. The lack of specific early diagnostic markers, coupled with the often asymptomatic progression of the tumor, means that many patients are diagnosed only after metastasis has occurred or experience recurrence following nephrectomy. Although targeted therapies such as tyrosine kinase inhibitors (TKIs) and immune checkpoint inhibitors (ICIs) have improved prognosis, their efficacy in metastatic settings is frequently limited by acquired resistance, treatment-related toxicities, and suboptimal response rates (6, 7). Therefore, identifying robust protective genes and elucidating their biological functions is essential for refining prognostic stratification and developing novel therapeutic approaches.

Trehalase (TREH) is a brush-border membrane enzyme primarily responsible for the hydrolysis of trehalose into two glucose molecules, playing a fundamental role in energy metabolism and glucose homeostasis (8). Beyond its canonical enzymatic function, the TREH has been implicated in critical cellular stress responses, including the regulation of autophagy and the mitigation of oxidative stress (9). Trehalose, the substrate of TREH, acts as a chemical chaperone and an mTOR-independent autophagy inducer, protecting cells against protein aggregation and oxidative damage (10). Consequently, the dysregulation of TREH expression may disrupt these protective mechanisms, potentially contributing to pathological states. In the context of oncology, the role of TREH is beginning to emerge. For instance, genome-wide association studies have identified specific TREH variants as susceptibility loci for glioblastoma, suggesting its potential involvement in tumorigenesis (11). In addition, because metabolic reprogramming, especially the “Warburg effect”, is a prominent feature of cancer, enzymes involved in glucose metabolism such as TREH have been increasingly intensively investigated for their potential as therapeutic targets (12). Despite these understandings, the specific expression profile, biological function, and prognostic value of TREH in clear cell renal cell carcinoma remain unexplored. Whether TREH acts as a tumor suppressor by regulating the metabolic or immune landscape of the kidney requires systematic elucidation.

In the present study, we employed a comprehensive multi-omics approach to systematically identify key protective genes in ccRCC. Based on the TCGA-KIRC cohort, we employed a multistep screening strategy including WGCNA and differential expression analysis. By further integrating LASSO and gradient boosting machine to optimize model performance, TREH was screened as an important and novel independent protective factor. We then further validated the clinical significance of TREH and explored its biological function at single-cell resolution using scRNA-seq analysis. Our integrated analysis revealed that *TREH* is specifically expressed in epithelial cells and that its downregulation is associated with a remodeled intercellular communication landscape. Notably, TREH-deficient epithelial cells were predicted to exhibit altered intercellular communication, with computational inference suggesting involvement of pro-angiogenic and growth-promoting pathways such as VEGFA, PLG, and APP. Moreover, validation assays confirmed that *TREH* overexpression significantly suppresses ccRCC growth and metastasis *in vitro* and *in vivo*, an effect concurrently associated with the inhibition of the EMT program. Therefore, TREH emerges as a candidate prognostic indicator and may warrant further exploration as a potential therapeutic target.

## Results

### Integrated bioinformatics screening identifies TREH as a novel prognostic protective gene in ccRCC

**Figure 1:**
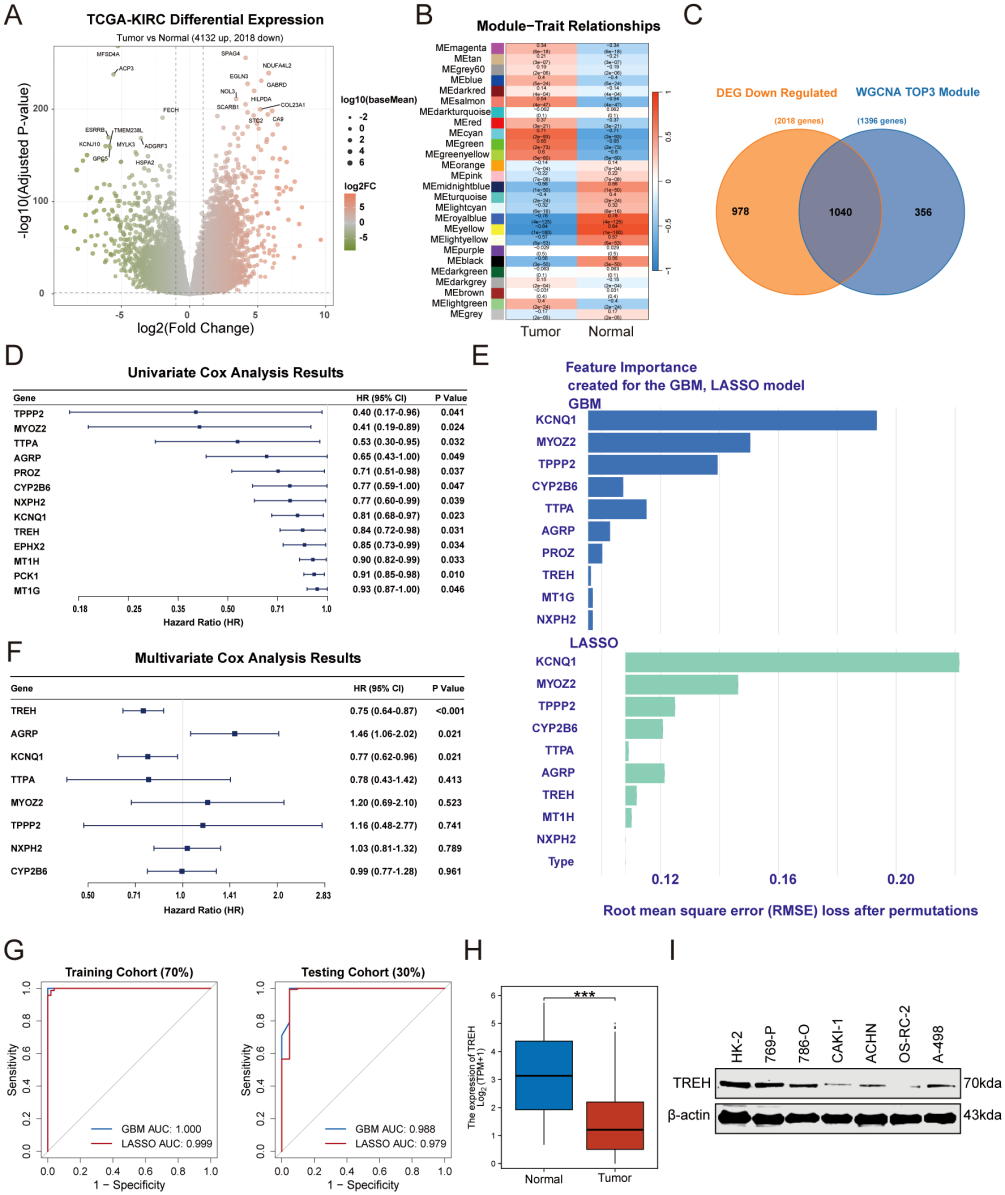
Integrated bioinformatic screening identifies TREH as a novel prognostic protective gene in ccRCC. **(A)** The TCGA-KIRC cohort was used to analyze the significantly differentially expressed genes. **(B)** WGCNA Identifies Gene Modules Negatively Correlated with Tumor Malignant Status in Multi-cohort Integrated Data. **(C)** Venn Diagram of the Intersection of Downregulated DEGs and WGCNA Anti-tumor Modules. **(D)** Univariate Cox regression analysis identified protective factors significantly associated with a good prognosis. **(E)** Two machine learning algorithms, LASSO and GBM, were used to screen predictive genes. **(F)** Multivariate Cox regression was used to analyze the association of independent protective factors. **(G)** ROC curves demonstrating the robust predictive performance of the GBM and LASSO machine learning models in both the training (70%, left panel) and internal testing (30%, right panel) cohorts. **(H)** The expression of TREH in KIRC tumor tissues was significantly lower than that in normal renal tissues in TCGA. **(I)** Validation of TREH expression in multiple ccRCC cell lines.

To systematically identify key protective genes in ccRCC, we performed an integrated bioinformatics analysis. Initially, differential expression analysis between tumor and normal tissues in the TCGA-KIRC cohort identified a total of 2018 significantly downregulated genes (**Figure 1A**). Concurrently, we employed WGCNA to map the landscape of gene modules driving clinical phenotypes. After rigorous data quality control via sample hierarchical clustering to remove outliers, we calculated the optimal soft-thresholding power to construct a robust scale-free co-expression network (**Supplementary Figure S1A**). Genes were subsequently partitioned into distinct color-coded modules based on topological overlap (**Supplementary Figure S1B**). The comprehensive lists of genes assigned to all identified modules are provided in **Supplementary Table S1**. Intriguingly, correlation analyses linking these modules to clinical traits highlighted three modules—lightyellow (R=-0.57, *p* < 0.001), royalblue (R=-0.78, *p* < 0.001), and yellow (R=-0.84, *p* < 0.01)—that were profoundly negatively correlated with tumor status, capturing a focused set of 1,396 genes (**Figure 1B**). To identify key prognostic genes, we first intersected the downregulated DEGs with the genes from the three critical WGCNA modules, yielding 1,040 candidates (**Figure 1C**). Subsequent univariate Cox regression analysis narrowed this pool down to 13 molecules significantly associated with a favorable prognosis (**Figure 1D**). This stable performance across cohorts confirmed the excellent generalization ability of our screening strategy. From these robust models, we extracted the top 10 predictive genes from each algorithm (**Figure 1E**). The intersection of these two feature lists yielded 8 consensus genes. Finally, multivariate Cox regression analysis of these 8 candidates revealed that Trehalase (*TREH*) emerged as the most significant independent protective factor, with its low expression robustly predicting poorer survival (Hazard Ratio<1, *p* < 0.001, **Figure 1F**).To further refine this prognostic signature rigorously, we randomly partitioned the TCGA-KIRC cohort into a training set (70%) and an internal testing set (30%), and employed two machine learning algorithms: LASSO and GBM. Both models demonstrated exceptional predictive accuracy in the training cohort (GBM AUC = 1.000; LASSO AUC = 0.999), and these results were successfully validated in the internal testing cohort (GBM AUC = 0.988; LASSO AUC = 0.979) (**Figure 1G**). Consistent with its identified protective role, TREH expression was significantly lower in KIRC tumor tissues compared to normal kidney tissues in the TCGA cohort (**Figure 1H**).The expression level of TREH in various renal cancer cell lines, including the normal renal cell line HK-2 and ccRCC cell lines (769-P, 786-O, CAKI-1, ACHN, OS-RC-2, A-498), was analyzed by Western blot. The results showed that compared with the normal renal cell line HK-2, TREH expression was significantly reduced in all ccRCC cell lines (**Figure 1I**), indicating that TREH is generally downregulated in renal cancer cells, which is consistent with the transcriptomic analysis results from the TCGA-KIRC cohort.

### TREH serves as an independent protective prognostic biomarker in ccRCC

**Figure 2:**
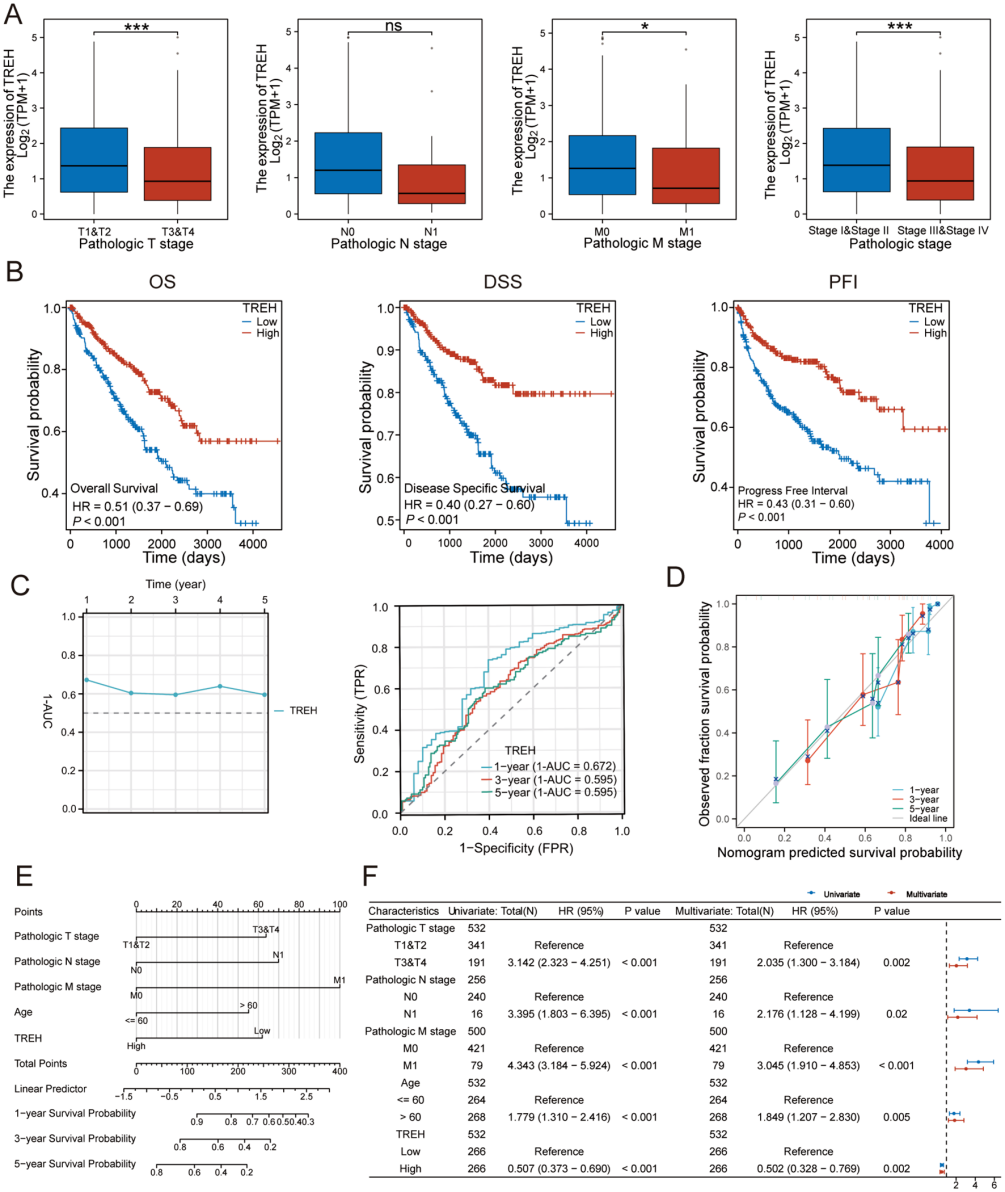
Clinical relevance and prognostic value validation of TREH in ccRCC. **(A)** Correlation analysis of TREH expression levels with clinicopathological characteristics in the TCGA-KIRC cohort. **(B)** Kaplan-Meier survival curves illustrating the association of TREH expression with OS, DSS, and PFI in ccRCC patients. **(C)** Time-dependent ROC curve analysis showing the AUC values of TREH for prognostic prediction at 1, 3, and 5 years. **(D)** Calibration plots demonstrating the predictive performance of the nomogram for 1-, 3-, and 5-year overall survival rates. **(E)** Prognostic nomogram integrating TREH expression with pathological T,N,M stages and Age for quantifying the survival risk of ccRCC patients. **(F)** Multivariate Cox regression analysis and forest plot revealing that TREH serves as an independent prognostic factor for poor OS in ccRCC. *p < 0.05, ***p < 0.001.ns: no significance.

The additional datasets GSE15642, GSE40435, and GSE53747 similarly verified TREH’s low expression in ccRCC (**Supplementary Figures S2A–C**). To assess the clinical relevance of TREH, we analyzed the correlation between TREH and key pathological stages in the TCGA-KIRC cohort. TREH expression was significantly lower in tumors with advanced pathological T stage (T3 and T4 vs. T1 and T2, *p* < 0.001) and distant metastases (M1 vs. M0, *p* < 0.05). Although the difference in lymph node status was not statistically significant (N1 vs. N0), a consistent trend of reduced TREH expression was observed in the high-risk group across all clinical stages (Stages III and IV vs. I and II, *p* < 0.001) (**Figure 2A**). The protective prognostic value of TREH was further substantiated by survival analysis. Kaplan-Meier curves demonstrated that patients with high TREH expression had significantly longer OS (*p* < 0.001), DSS (*p* < 0.001), and PFI (*p* < 0.001) compared to those with low expression (**Figure 2B**). Next, we evaluated the predictive accuracy of TREH. Time-dependent AUC analysis confirmed that its predictive power was stable over 5 years. Similarly, time-dependent ROC curves showed 1-AUC values above 0.5 at 1, 3, and 5 years (0.672,0.595 and 0.595, respectively), a pattern that suggests its role as a protective biomarker, with high expression associated with poor prognosis (**Figure 2C**). In this model, lower TREH expression contributed a higher point score, which corresponded to a worse predicted survival probability. The calibration plots for 1-, 3-, and 5-year survival showed good agreement between the nomogram’s predictions and actual observed outcomes (**Figure 2D**). To facilitate clinical translation, a prognostic nomogram integrating TREH expression with Pathologic T,N,M stages and Age was constructed (**Figure 2E**). Finally, multivariate Cox regression analysis, which included TREH expression and significant with Pathologic T,N,M stages and Age, confirmed that low TREH expression remained an independent risk factor for poor OS after adjusting for these confounders (**Figure 2F**).

### Single-cell transcriptomic atlas reveals TREH’s specific expression in epithelium and its association with a protective immune microenvironment

**Figure 3:**
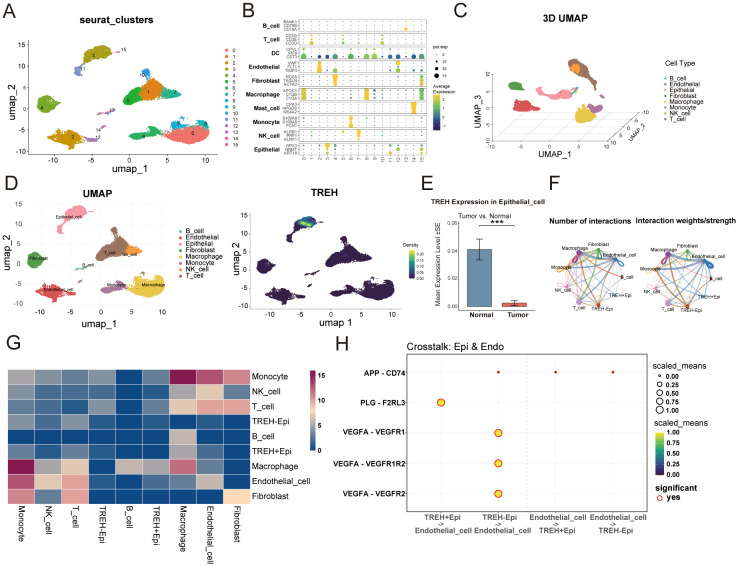
Single-cell landscape suggests association of TREH deficiency with pro-angiogenic signaling predictions. **(A)** Cell population clustering analysis of scRNA-seq data (GSE242299: 8 tumor vs. 8 normal samples) based on UMAP projection. **(B)** Marker gene bubble plot displaying canonical marker genes for the annotation of cell clusters into major cell lineages. **(C)** 3D UMAP visualization of the major cell types in ccRCC tissues. **(D)** Nebulosa density map illustrating the spatial distribution of TREH-expressing cells. **(E)** Bar plot comparing TREH expression levels in epithelial cells isolated from adjacent normal and tumor tissues of ccRCC. **(F)** Cell communication network plot depicting the quantity and strength of intercellular interactions between TREH-high (TREH^+^Epi) and TREH-low (TREH^-^Epi) epithelial cell subsets with other cells in the tumor microenvironment. **(G)** Differential interaction heatmap showing the global shifts in signaling strength between the two epithelial subsets and other cell types. **(H)** Predicted ligand-receptor pairs from CellChat analysis, suggesting potential differences in crosstalk patterns between TREH-low and TREH-high epithelial cells. These bioinformatic predictions are hypothesis-generating and require experimental validation.

To dissect the cellular basis of TREH’s function at single-cell resolution, we analyzed a ccRCC scRNA-seq dataset. Unsupervised clustering revealed 16 distinct cell populations (**Figure 3A**), which were subsequently annotated into major lineages, including epithelial cells, T cells, macrophages, endothelial cells, and fibroblasts—based on canonical marker gene expression (**Figure 3B**). The annotated cellular landscape was visualized in a 3D UMAP plot (**Figure 3C**). Examination of TREH expression across the atlas using Nebulosa, indicated its predominant and specific expression within the epithelial cell compartment (**Figure 3D**). Consistently with bulk RNA-seq findings, epithelial cells derived from normal adjacent tissues exhibited significantly higher TREH expression compared to those from tumor tissues (**Figure 3E**). We next sought to understand how TREH expression in epithelial cells influences intercellular communication. Epithelial cells were stratified into TREH^+^Epi (high expression) and TREH^-^Epi (low expression) subgroups (**Figure 3F**). ccRCC is notoriously characterized by robust angiogenesis and hypervascularization. Accordingly, decoding the intercellular cross-talk between epithelial subsets and other cell types in the vicinity is crucial for understanding microenvironmental remodeling during tumor progression. CellChat analysis suggested that the loss of TREH was associated with a marked reconfiguration of these signaling intensities (**Figure 3G**). Detailed analysis of the predicted ligand-receptor pairs highlighted a distinct transition in communication strategies (**Figure 3H**). While TREH+ epithelial cells appeared to maintain baseline interactions characterized by structural signaling pathways, computational modeling suggested that TREH- epithelial cells exhibited predicted more intensive and potentially aggressive communication networks. Notably, this subset was predicted to involve potent signaling primarily through the VEGF (e.g., VEGFA-KDR, VEGFA-FLT1) and PLG (PLG-F2RL3) pathways, alongside enriched APP (APP-CD74) interactions. These bioinformatic inferences suggest that TREH deficiency may reprogram epithelial cells to actively modulate the surrounding microenvironment, potentially creating a niche conducive to malignant progression. All single-cell and CellChat analyses presented in this section are predictive and intended to generate hypotheses. They do not constitute functional validation of intercellular signaling.

### TREH inhibits the proliferation of KIRC tumor *in vitro* and *in vivo*

**Figure 4:**
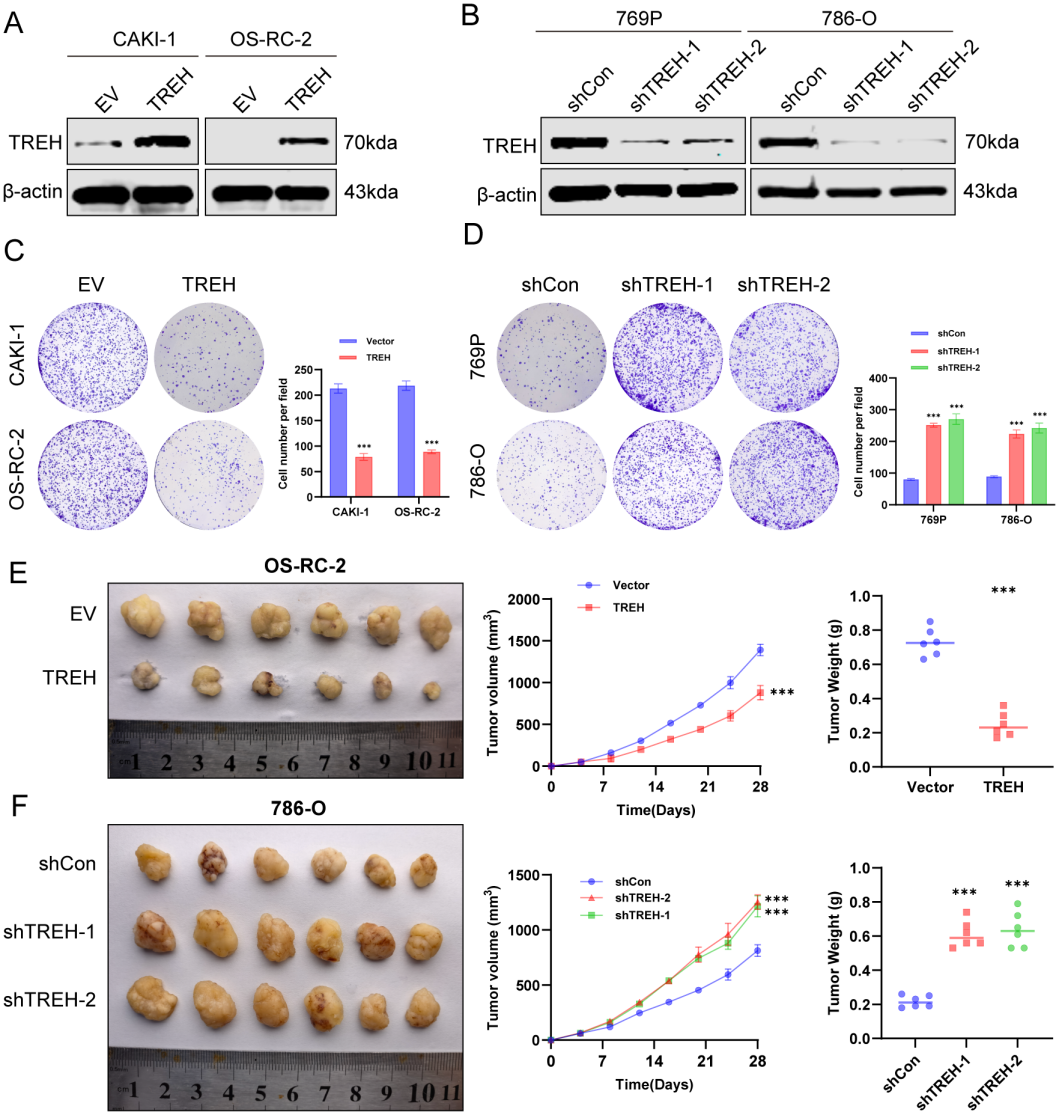
*In vitro* and *in vivo* experiments verified the effect of TREH on the proliferation function of ccRCC. **(A)** Protein expression of TREH in CAKI-1 and OS-RC-2 cell lines after TREH overexpression. **(B)** Protein expression of TREH in 769P and 786-O cell lines after TREH knockdown. **(C)** Colony formation assay of cell lines after TREH overexpression. **(D)** Colony formation assay of cell lines after TREH knockdown. **(E)** Tumor growth rate and volume changes in BALB/c-nu mice with overexpressing of TREH **(H)** With knockdown TREH(n=6). *p < 0.05, **p < 0.01, ***p < 0.001.

The protein expression level of TREH was detected at the cellular level previously. Among them, CAKI-1 and OS-RC-2 cell lines showed the low TREH expression and 786-O and 769P cell lines showed the high TREH expression, and we chose to establish stable TREH overexpression and knockdown models for further functional analysis (**Figures 4A, B**). We evaluated its effect on ccRCC cell proliferation using clone formation assay. Overexpression of TREH significantly inhibited the proliferation of CAKI-1 and OS-RC-2 cells, while inhibition of TREH significantly promoted the proliferation of 769P and 786-O cells (**Figures 4C, D**). The animal model was subcutaneous transplanted tumor experiment in nude mice. Tumor growth was significantly inhibited in OS-RC-2 with TREH overexpression compared to control (**Figure 4E**), whereas knockdown of TREH in 786-O promoted tumor growth (**Figure 4F**).

### TREH inhibits the migration and invasion of KIRC tumors

**Figure 5:**
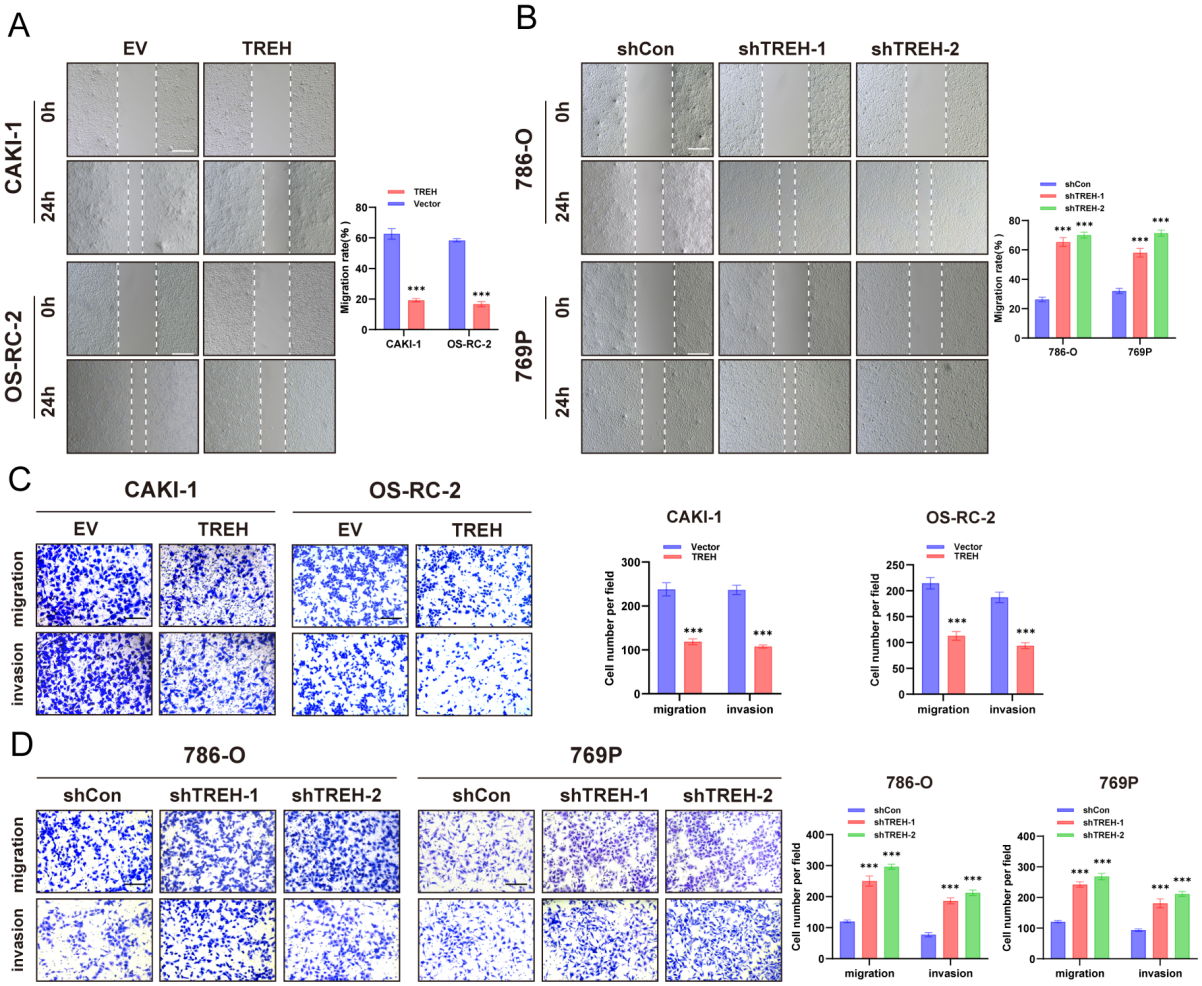
*In vitro* assays validated the impact of TREH on the migratory and invasive capacities of ccRCC. Cell migration and invasion abilities were determined by Transwell and scratch assays using TREH-overexpressing CAKI-1 and OS-RC-2 cell lines **(A, C)** and TREH-silenced 786-Oand 769P cell lines **(B, D)**. Scale bars: 50 μm. ***p < 0.001.

TREH overexpression markedly reduced the migration and invasion abilities of ccRCC cell lines (**Figure 5A**), while silencing of TREH yielded contrasting outcomes (**Figure 5B**). Furthermore, wound healing assays further revealed that TREH overexpression significantly reduced the migration potential of these cells (**Figure 5C**), with TREH silencing exhibiting the opposite effect (**Figure 5D**). These collective findings underscore that TREH plays a pivotal role in inhibit the migration and invasion of ccRCC cells.

### Functional enrichment and pathway activity analysis reveals TREH’s association with metabolic processes and EMT suppression

**Figure 6:**
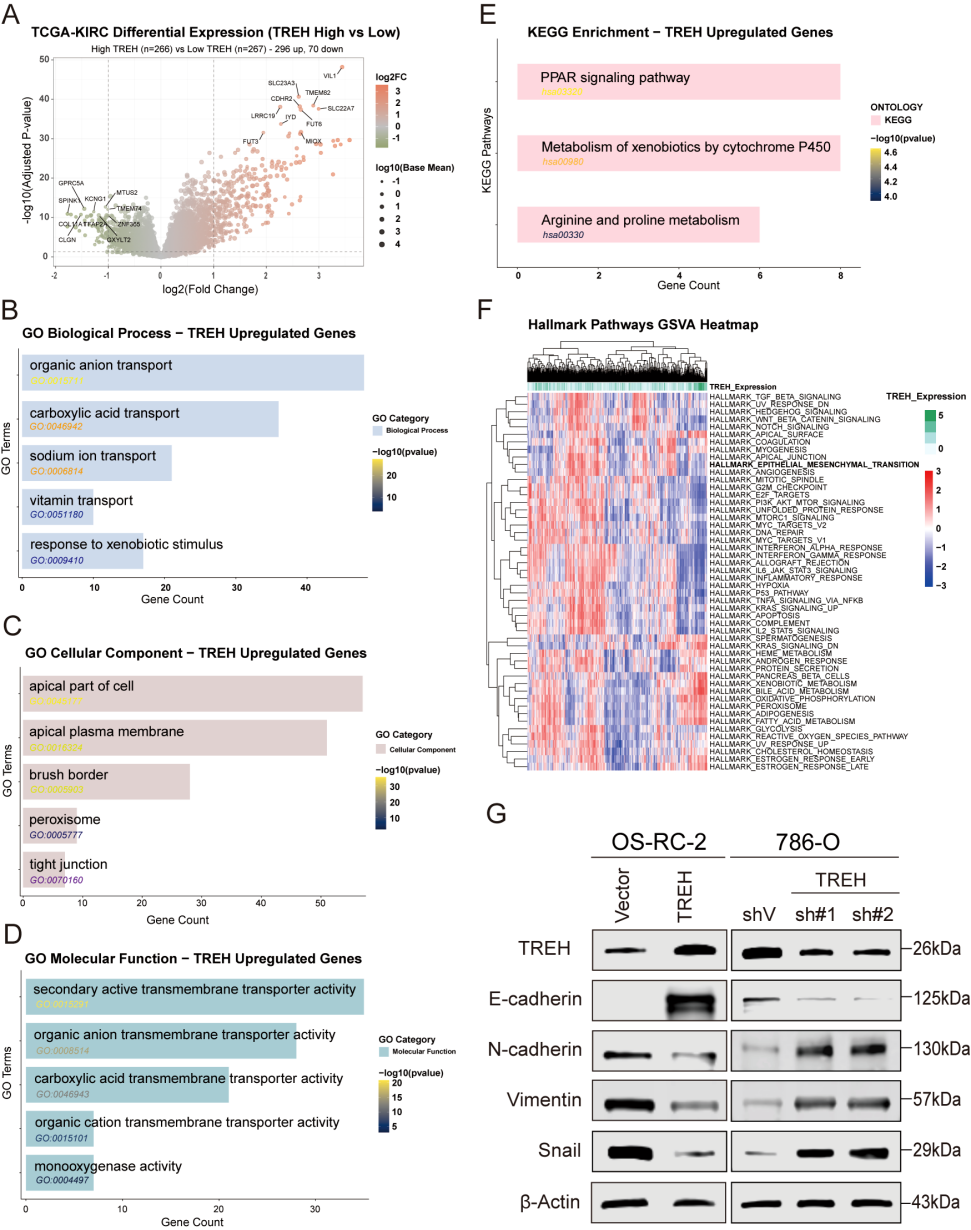
Bioinformatic analysis and experimental validation of TREH as a negative regulator of EMT in KIRC. **(A)** Volcano plot showing the distribution of DEGs between TREH-high and -low expression groups in the TCGA-KIRC cohort. **(B–D)** GO enrichment analysis of TREH-up-regulated genes. Significant enrichment in **(B)** biological processes, **(C)** cellular components, and **(D)** molecular functions is shown. **(E)** KEGG pathway analysis revealing that TREH-related genes were mainly enriched in pathways. **(F)** GSVA heatmap illustrating the differential activity of signaling pathways correlated with TREH expression. **(G)** TREH overexpression in OS-RC-2 cells and TREH knockdown in 786-O cells were used to verify the regulatory role of TREH in EMT by Western Blot.

Using the TCGA-KIRC dataset, we performed differential expression analysis on high- and low-TREH tumors to identify potential functional impacts (**Figure 6A**) displays the resulting gene expression differences via a volcano plot. For genes upregulated in the high-TREH group, functional enrichment was analyzed. GO results emphasized transport pathways (BP: carboxylic acid and organic anion transport), structural locations (CC: cell apical part, tight junctions), and specific activities (MF: transmembrane transport) (**Figures 6B–D**). Additionally, KEGG pathway evaluation showed an upregulation of metabolic processes, specifically regarding drug metabolism and xenobiotic processing by cytochrome P450 (**Figure 6E**). To further explore the potential downstream effects of the altered intercellular communication observed in our CellChat analysis, we evaluated the activity of broad oncogenic pathways in relation to TREH expression using GSVA with Hallmark gene sets. Intriguingly, the Epithelial-Mesenchymal Transition (EMT) pathway emerged as the most significantly and robustly negatively correlated with TREH levels (logFC=-0.076, adj.P.Val =1.08^e-13^, B statistic=21.77) (**Figure 6F**). This finding, coupled with the previously inferred pro-tumorigenic cross-talk, suggests a potential link between TREH deficiency and a more mesenchymal, aggressive phenotype. Specifically, these bioinformatic predictions raise the hypothesis that TREH loss may contribute to EMT-like changes. Independent of these predictions, protein-level analysis of key EMT markers confirmed a significant inverse correlation between TREH expression and mesenchymal markers (**Figure 6G**), supporting the association of TREH with a less aggressive cellular state.

## Discussion

By integrating bulk and single-cell transcriptomics with machine learning and *in vitro*/*in vivo* functional assays, this study identifies TREH as a potential independent protective biomarker and candidate tumor suppressor in ccRCC. Our findings indicate that low TREH expression is associated with EMT activation and with computational predictions of a pro-angiogenic microenvironment (VEGF/PLG/APP pathways). A prognostic nomogram incorporating TREH showed improved predictive accuracy. These results suggest that the role of glucose metabolism enzymes in ccRCC progression has been previously underestimated.

TREH is a classic brush-border membrane enzyme primarily responsible for trehalose hydrolysis, and its biological functions have been previously reported mainly in glucose homeostasis, metabolic diseases, and cellular stress responses. In terms of metabolic regulation, TREH-mediated trehalose catabolism is critical for maintaining systemic glucose balance, and genetic variations in TREH have been associated with the risk of type 2 diabetes in human populations (13, 14). In cellular stress responses, TREH deficiency leads to trehalose accumulation, which in turn regulates autophagy and oxidative stress resistance by acting as a chemical chaperone and mTOR-independent autophagy inducer (9, 10). In the field of oncology, research on TREH remains limited and fragmented: a genome-wide association study identified specific TREH variants as susceptibility loci for glioblastoma, suggesting its potential involvement in tumorigenesis (11), but no functional validation was performed to confirm whether TREH acts as a driver gene or just a disease-associated gene in glioblastoma. To date, there have been no reports on the expression profile, biological function, and clinical significance of TREH in urinary system tumors, especially ccRCC. Our study expands the research landscape of TREH in oncology: unlike its merely associative role in glioblastoma, we confirmed that TREH has a direct functional tumor-suppressive effect in ccRCC through gain-and loss-of-function experiments in multiple cell lines and *in vivo* xenograft models. Meanwhile, consistent with its classical metabolic regulatory function, our KEGG enrichment analysis showed that TREH-upregulated genes are significantly enriched in metabolic pathways such as drug metabolism and xenobiotic processing by cytochrome P450, indicating that TREH still exerts metabolic regulatory effects in ccRCC. More importantly, we discovered a non-canonical function of TREH in regulating EMT and tumor microenvironment intercellular communication, which complements the understanding of the multifunctional properties of TREH and provides a new perspective for exploring the role of metabolic enzymes in tumor progression.

ccRCC is a malignancy characterized by profound intratumoral heterogeneity and metabolic reprogramming, with EMT and abnormal angiogenesis being two core mechanisms driving its invasion, metastasis, and therapy resistance (15–17). EMT is a key cellular process by which epithelial tumor cells acquire mesenchymal characteristics, and its activation in ccRCC is closely associated with distant metastasis and poor prognosis, and is regulated by multiple signaling pathways such as TGF-β and VEGF (15, 18). Abnormal angiogenesis, a typical pathological feature of ccRCC, is driven by the intensive crosstalk between tumor cells and stromal cells in the microenvironment, and the VEGF signaling pathway is the core target of clinical anti-angiogenic therapy for ccRCC (16, 19, 20). Our study reveals a new regulatory link between metabolic enzyme dysregulation and these two classic oncogenic mechanisms in ccRCC: GSVA analysis confirmed a robust negative correlation between TREH expression and EMT pathway activity, and Western blot experiments further validated that TREH overexpression significantly upregulates the epithelial marker E-cadherin and downregulates mesenchymal markers (N-cadherin, Vimentin) and the EMT transcription factor Snail, while TREH knockdown yields the opposite effect. This finding identifies TREH as a novel negative regulator of EMT in ccRCC, filling the research gap in the regulation of EMT by disaccharide metabolic enzymes in ccRCC, and revealing a new cross-talk between metabolic reprogramming and EMT in this cancer type. At the single-cell level, our CellChat analysis provided computational predictions that TREH-deficient epithelial cells may engage in more intensive intercellular communication with endothelial cells and macrophages, potentially involving VEGF, PLG, and APP pathways. These predictions are hypothesis-generating and await experimental testing. Previous studies have largely focused on stromal cells in ccRCC angiogenesis; our analysis raises the possibility that TREH-low epithelial cells could also actively contribute to microenvironmental remodeling (17, 21). Collectively, these results confirm that TREH deficiency coordinately drives ccRCC progression through the intrinsic activation of EMT and extrinsic remodeling of the pro-angiogenic microenvironment, and this dual mechanism reveals the unique regulatory role of TREH in ccRCC, which is different from its function in other physiological and pathological processes.

The clinical value of our study is reflected in the identification of TREH as a robust prognostic biomarker and the construction of a more accurate prognostic nomogram for ccRCC. Currently, clinical prognostic stratification of ccRCC mainly relies on traditional TNM staging and histological grading, but these systems fail to capture the complex biological heterogeneity of tumors, leading to inaccurate risk assessment for some patients, especially early-stage patients with occult metastatic potential (22). Although some gene signature-based prognostic models have been developed for ccRCC, most are constructed based on bulk transcriptome data without single-cell level validation, and lack the integration of metabolic biomarkers, limiting their clinical application (22). Our study confirmed through univariate and multivariate Cox regression analyses that TREH is an independent prognostic factor for ccRCC, and its prognostic value is stable across multiple datasets (TCGA-KIRC, GSE15642, GSE40435, GSE53747). Time-dependent ROC curve analysis showed that TREH has a sustained predictive ability for 1-, 3-, and 5-year overall survival of ccRCC patients. More importantly, the nomogram exhibited promising predictive performance in our retrospective analysis. Calibration plots showed good agreement between predicted and observed survival. However, prospective multi-center validation is needed before clinical application. If confirmed, this nomogram could potentially aid in risk stratification, particularly for identifying patients with low TREH expression who may warrant closer follow-up. In addition, our mechanistic findings also provide potential therapeutic strategies for ccRCC: patients with low TREH expression have an activated VEGF signaling pathway in the tumor microenvironment, suggesting that these patients may benefit more from anti-angiogenic therapy targeting the VEGF pathway; meanwhile, inducing TREH overexpression or mimicking its biological functions may become a new therapeutic approach to reverse EMT and inhibit tumor angiogenesis in ccRCC.

Despite the significant findings of this study, several limitations need to be acknowledged, which also point out the direction for subsequent research. First, although we confirmed that TREH regulates EMT and pro-angiogenic signaling in ccRCC, the precise molecular link between TREH’s enzymatic activity (trehalose hydrolysis) and these regulatory effects remains unclear. It is unknown whether the tumor-suppressive effect of TREH depends on its classical catalytic function (e.g., regulating glucose metabolism or trehalose accumulation) or its non-catalytic moonlighting function (23, 24). Additionally, all intercellular communication inferences from CellChat are correlational and computational in nature; they do not constitute functional evidence of ligand-receptor activation *in vivo*. Thus, we have not established a direct causal relationship between TREH’s trehalase activity and the observed phenotypic changes (EMT, migration, invasion). Subsequent studies will construct catalytically inactivated TREH mutants to perform rescue experiments, and combine metabolite profiling to clarify the role of TREH’s enzymatic activity in ccRCC progression. Second, we have not explored the upstream regulatory mechanism of TREH downregulation in ccRCC. ccRCC is characterized by frequent VHL gene mutations, which lead to abnormal activation of the HIF pathway and subsequent metabolic reprogramming and angiogenesis (17, 21); it is necessary to further verify whether the VHL-HIF pathway regulates TREH expression at the transcriptional or epigenetic level through ChIP-seq, methylation sequencing, and other experiments. Third, although we validated the prognostic value of TREH in multiple public datasets, the validation was based on retrospective cohorts; further prospective multi-center clinical studies with larger sample sizes are needed to confirm the clinical utility of TREH as a prognostic biomarker, and to develop standardized IHC detection kits for TREH to facilitate its clinical translation. Fourth, our *in vivo* experiments only used subcutaneous xenograft models, which cannot fully simulate the tumor microenvironment and metastatic process of ccRCC; subsequent studies will use orthotopic xenograft models and tail vein injection metastasis models to further verify the effect of TREH on ccRCC invasion and metastasis in a more physiological context. Finally, we only explored the role of TREH in regulating the crosstalk between epithelial cells and other stromal cells at the transcriptomic level; subsequent studies will use co-culture systems, spatial transcriptomics, and immunofluorescence staining to further validate the intercellular communication mechanism mediated by TREH deficiency at the cellular and tissue levels (25, 26).

In conclusion, our study identifies TREH as a candidate prognostic biomarker and suggests its potential role as a tumor suppressor in ccRCC. Our findings indicate that low TREH expression correlates with EMT activation and with computational predictions of a pro-angiogenic microenvironment. Further mechanistic studies are required to determine whether these effects are directly mediated by TREH’s enzymatic activity. This study expands the understanding of the biological functions of TREH in oncology, reveals a new cross-talk between disaccharide metabolic enzyme dysregulation and ccRCC oncogenic mechanisms, and provides a novel metabolic biomarker for the prognostic stratification of ccRCC patients. Meanwhile, our findings suggest that targeting the TREH-mediated metabolic-microenvironmental axis may become a new strategy for the individualized treatment of ccRCC, which has important theoretical and clinical significance. Future research will focus on dissecting the precise molecular mechanism of TREH in ccRCC, verifying its clinical utility in prospective cohorts, and exploring the potential of TREH as a therapeutic target, in order to translate these basic research findings into clinical practice and improve the diagnosis and treatment level of ccRCC.

## Data Availability

The original contributions presented in the study are included in the article/**Supplementary Material**. Further inquiries can be directed to the corresponding author.
