# Supplementary material for: Integrated bulk and single-cell transcriptomics reveals TREH as a novel protective biomarker and prognostic predictor in clear cell renal cell carcinoma
Source: Front Oncol. 2026 May 11;16:1808369. doi: 10.3389/fonc.2026.1808369 (PMC13199097; doi:10.3389/fonc.2026.1808369)
Supplement: Supplementary file 1 [file DataSheet1.docx]

Supplementary Materials


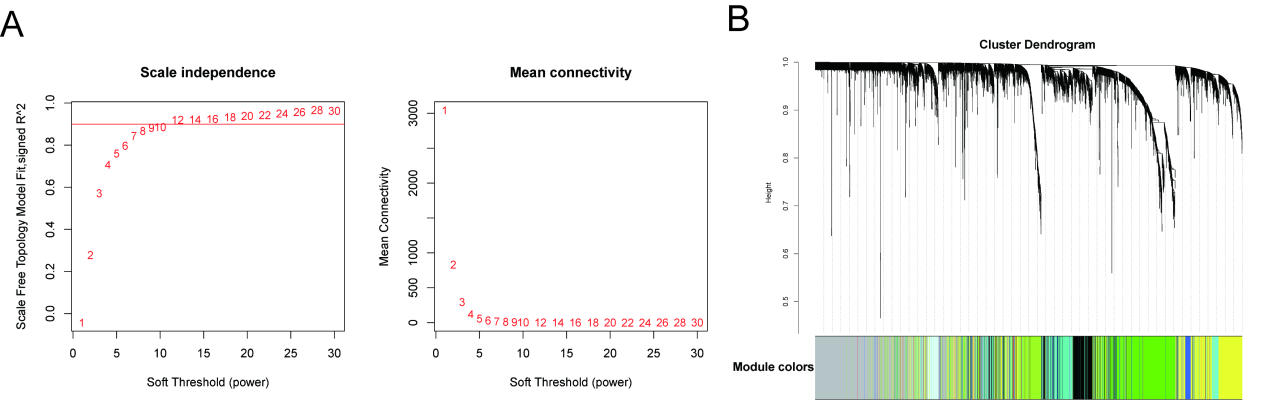


**Supplementary Figure S1. Construction of the weighted gene co-expression network.**

**(A)** Analysis of network topology for various soft-thresholding powers. The left panel displays the scale-free fit index (R-squared) as a function of the soft-thresholding power (β). The right panel shows the mean connectivity for different soft-thresholding powers. The optimal power was selected to ensure a scale-free network. **(B)** Gene clustering dendrogram based on topological overlap. Each branch in the tree represents an individual gene, and the colored band below indicates the specific co-expression modules identified by the dynamic tree cut algorithm.


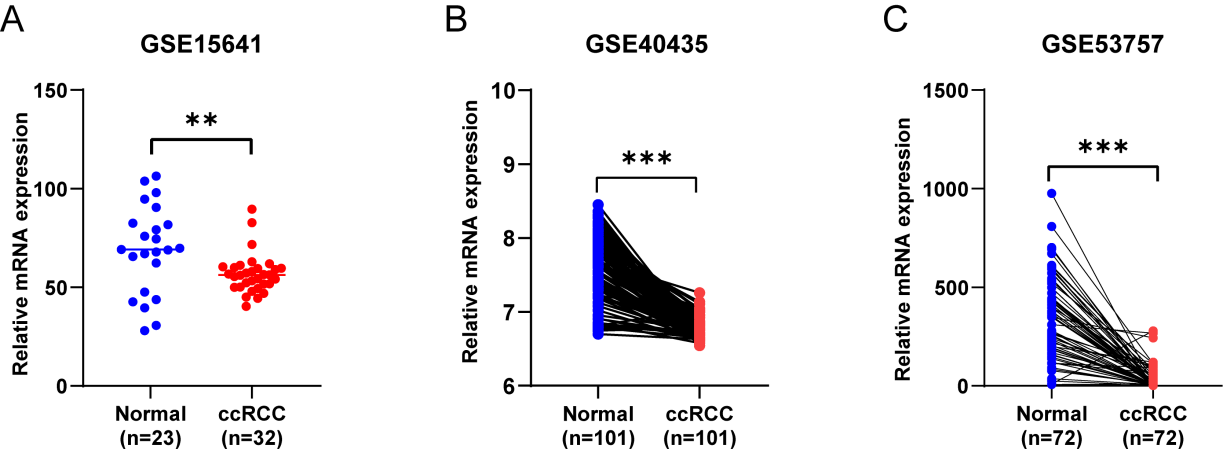


**Supplementary Figure S2.** GEO databases were used to analyze the expression of TREH in ccRCC tissues.

**Supplementary Tables**

**Supplementary Table 1. List of genes resulting from WGCNA**
